# Supplementary material for: The rise and fall of the ancient northern pike master sex-determining gene
Source: eLife. 2021 Jan 28;10:e62858. doi: 10.7554/eLife.62858 (PMC7870143; doi:10.7554/eLife.62858)
Supplement: Transparent reporting form [file elife-62858-transrepform.docx]

***eLife’s* transparent reporting form**

We encourage authors to provide detailed information *within their submission* to facilitate the interpretation and replication of experiments. Authors can upload supporting documentation to indicate the use of appropriate reporting guidelines for health-related research (see [EQUATOR Network](http://www.equator-network.org/%20)), life science research (see the [BioSharing Information Resource](https://biosharing.org/)), or the [ARRIVE guidelines](http://www.plosbiology.org/article/info:doi/10.1371/journal.pbio.1000412) for reporting work involving animal research. Where applicable, authors should refer to any relevant reporting standards documents in this form.

If you have any questions, please consult our Journal Policies and/or contact us: [editorial@elifesciences.org](mailto:editorial@elifesciences.org).

**Sample-size estimation**

- You should state whether an appropriate sample size was computed when the study was being designed
- You should state the statistical method of sample size computation and any required assumptions
- If no explicit power analysis was used, you should describe how you decided what sample (replicate) size (number) to use

Please outline where this information can be found within the submission (e.g., sections or figure legends), or explain why this information doesn’t apply to your submission:

The sample size for each species analyzed for sex linkage can be found in Table 1. We did not perform power analysis to determine the sample size as one challenge of our study was to acquired fish samples in the wild from three continents with phenotypic sex confidently determined. As a result, sample sizes were determined based on availability/opportunity. We are aware that for two species (*Esox cisalpinus* and *Esox aquitanicus*), we have very low sample size, which is due to the fact that these two species were only recently identified as separate species from *Esox lucius* and we had to rely on museum samples with clear sex phenotype information to have confidence in species identification. This limitation is stated in the result section “Sex-linkage of *amhby* in the Esocidae lineage”.

**Replicates**

- You should report how often each experiment was performed
- You should include a definition of biological versus technical replication
- The data obtained should be provided and sufficient information should be provided to indicate the number of independent biological and/or technical replicates
- If you encountered any outliers, you should describe how these were handled
- Criteria for exclusion/inclusion of data should be clearly stated
- High-throughput sequence data should be uploaded before submission, with a private link for reviewers provided (these are available from both GEO and ArrayExpress)

Please outline where this information can be found within the submission (e.g., sections or figure legends), or explain why this information doesn’t apply to your submission:

Information on experiment design and sample size for all datasets can be found in the Material and Methods section. We didn’t not exclude any sample.

All sequencing data (RAD-Seq, Pool-Seq and draft genome sequencing) for this study can be found on NCBI under the common project number PRJN634624.

**Statistical reporting**

- Statistical analysis methods should be described and justified
- Raw data should be presented in figures whenever informative to do so (typically when N per group is less than 10)
- For each experiment, you should identify the statistical tests used, exact values of N, definitions of center, methods of multiple test correction, and dispersion and precision measures (e.g., mean, median, SD, SEM, confidence intervals; and, for the major substantive results, a measure of effect size (e.g., Pearson's r, Cohen's d)
- Report exact p-values wherever possible alongside the summary statistics and 95% confidence intervals. These should be reported for all key questions and not only when the p-value is less than 0.05.

Please outline where this information can be found within the submission (e.g., sections or figure legends), or explain why this information doesn’t apply to your submission:

Statistical methods can be found in the Material and Methods section. Detailed statistical results are reported within the Results section with the test performed and exact P values. For the RAD-Seq analyses, Bonferroni corrections were performed to adjust P-values according to marker numbers in each dataset.

(For large datasets, or papers with a very large number of statistical tests, you may upload a single table file with tests, Ns, etc., with reference to sections in the manuscript.)

**Group allocation**

- Indicate how samples were allocated into experimental groups (in the case of clinical studies, please specify allocation to treatment method); if randomization was used, please also state if restricted randomization was applied
- Indicate if masking was used during group allocation, data collection and/or data analysis

Please outline where this information can be found within the submission (e.g., sections or figure legends), or explain why this information doesn’t apply to your submission:

Samples were allocated into males and females based on phenotypic traits. The criteria for group allocation for each species can be found in the Material and Methods section.

**Additional data files (“source data”)**

- We encourage you to upload relevant additional data files, such as numerical data that are represented as a graph in a figure, or as a summary table
- Where provided, these should be in the most useful format, and they can be uploaded as “Source data” files linked to a main figure or table
- Include model definition files including the full list of parameters used
- Include code used for data analysis (e.g., R, MatLab)
- Avoid stating that data files are “available upon request”

Please indicate the figures or tables for which source data files have been provided:

We provided source data files, including output files from PSASS and RADSex pipeline (details for running these pipelines can be found in the Material and Methods section) and R scripts for generating figures for Figure 3 and Figure 4 and FigureS4 and Figure S5.

For Figure 3:

Figure 3-source data 1: Distribution of RADsex markers of E. masquinongy from a Quebec population with a minimal marker depth of 10 reads

Figure 3-source data 2:Distribution of RADsex markers of E. masquinongy from a Iowa population with a minimal marker depth of 10 reads

Figure 3-source data 3:Distribution of RADsex markers of N.hubbsi a minimal marker depth of 10 reads

Figure 3-source data 4: Distribution of RADsex markers of U.pygmaea a minimal marker depth of 10 reads

Figure 3-source data 5: Distribution of RADsex markers of D.pectoralis a minimal marker depth of 10 reads

Figure 3-source code 1: R Script to generate Figure 3

For Figure 4:

Figure 4-source data 1: Poolseq comparison of sex specific SNPs in windows of 50kb between males and female from a European population of E.lucius

Figure 4-source data 2: Poolseq comparison of sex specifc converage in windows of 1kb between males and female from a European population of E.lucius

Figure 4-source data 3: Poolseq comparison of sex specific SNPs in windows of 50kb between males and female from a North American population of E.lucius

Figure 4-source data 4: Poolseq comparison of sex specifc converage in windows of 1kb between males and female from a North American population of E.lucius

Figure 4-source data 5: E.lucius chromosome length file for the R script

Figure 4-source code 1: R script to generate Figure 4

For Appendix 1-figure3:

Source Code File 1: R script to generate Appendix 1-figure 3.R

Source data 1: Poolseq comparison of sex specifc converage in windows of 1kb between males and female from a North American population of E.lucius

Source data 2: Distribution of RADsex markers of a Canadian population of E.lucius with a minimal marker depth of 10 reads

Source data 3: Distribution of RADsex markers of a second Canadian population of E.lucius with a minimal marker depth of 10 reads

For Appendix 1-figure2:

Source data 4: Poolseq comparison of sex specific SNPs in windows of 50kb between males and female from a Canadian population of E.lucius

Source data 5: Poolseq comparison of sex specific SNPs in windows of 50kb between males and female from an Iowa population of E.masquinongy

Source data 6: Poolseq comparison of sex specifc converage in windows of 1kb between males and female from an Iowa population of E.masquinongy

Source data 7: E.masquinongy chromosome length file for the R script
